# Molecularly Imprinted Polymer-Quantum Dot Materials in Optical Sensors: An Overview of Their Synthesis and Applications

**DOI:** 10.3390/bios11030079

**Published:** 2021-03-13

**Authors:** Myriam Díaz-Álvarez, Antonio Martín-Esteban

**Affiliations:** Departamento de Medio Ambiente y Agronomía, INIA, Carretera de A Coruña km 7.5, 28040 Madrid, Spain; mdalvarez@inia.es

**Keywords:** molecularly imprinted polymer, quantum dots, fluorescence sensor, nanosensor, fluorescence quenching

## Abstract

In the last decades analytical methods have focused on the determination of target analytes at very low concentration levels. This has been accomplished through the use of traditional analytical methods that usually require high reagent consumption, expensive equipment and long pretreatment steps. Thus, there is a demand for simple, rapid, highly selective and user-friendly detection procedures. Quantum dots (QDs) are semiconductor fluorescent nanomaterials with unique optoelectronic properties that have shown great potential for the development of fluorescence probes. Besides, the combination of QDs with molecularly imprinted polymer (MIPs), synthetic materials with selective recognition, have been proposed as useful materials in the development of optical sensors. The resulting MIP-QDs optical sensors integrate the advantages of both techniques: the high sensitivity of QDs-based fluorescence sensors and the high selectivity of MIPs. This review gives a brief overview of the strategies for the synthesis of MIPs-QDs based optical sensors, highlighting the modifications in the synthesis procedure that improve the sensor performance. Finally, a revision of recent applications in sensing and bioimaging is presented.

## 1. Introduction

In the last few decades, many countries have restricted the maximum levels of pesticides and pharmaceutical residues permitted in food and the environment due to the growing concern about food safety and environmental protection. Besides, the detection of clinically important analytes and biomarkers at trace levels has been a main goal in the biomedical field. Thus, analytical methods have focused on the determination of target analytes at very low concentration levels. This has been achieved by using analytical instrumentation, mainly high-performance liquid chromatography (HPLC) and gas chromatography (GC) coupled with mass spectrometry because of their high sensitivity and identification capability. However, these methods usually involve long operation time, high cost, long pretreatment steps and high reagent consumption. Therefore, the development of simple, rapid, highly selective and user-friendly detection strategies has been required [1].

Quantum dots (QDs) are semiconductor fluorescent nanomaterials that have unique electronic and optical properties due to the quantum mechanics rules. Some of these outstanding optoelectronic characteristics include high quantum yields, high photostability, and broad absorption spectra together with narrow photoluminescent emission spectra. Because QDs present an emission band narrower than organic dyes, detections performed by QDs are less subjected to interferences. Besides, it has been evidenced that QDs are much more stable than organic fluorescent dyes [2]. As a result of such attractive properties, QDs have emerged as a powerful analytical tool leading to the introduction of a new category of fluorescent probes that have shown great potential for biological, biomedical, and environmental monitoring [3,4,5].

Fluorescence sensors have become the most widespread optical technique due to the low detection limits that can be achieved and the simplicity of the format; however, QDs-based probes have been reported to offer low selectivity because of the quenching phenomena occurring in the presence of certain substances (analytes), but also in the presence of sample constituents. Improvements in selectivity of QDs for a certain analyte can be successfully achieved by modification of QDs surfaces with target-specific materials such as molecularly imprinted polymers (MIPs), thus combining the high selectivity of MIPs and the substantial (quenchable) fluorescence of QDs.

MIPs are synthetic materials with recognition sites artificially designed for selectively rebinding a target molecule in preference to other related compounds. MIPs are prepared by the copolymerization of a cross-linker with a suitable monomer in the presence of a template molecule. When the polymerization has finished, the template molecule is extracted and binding sites with shape, size, and functionalities complementary to the target analyte are created within the polymeric matrix. MIPs have many advantages including good selectivity, high physical robustness, thermal stability, and inexpensive and easy preparation [6,7].

**Figure 1:**
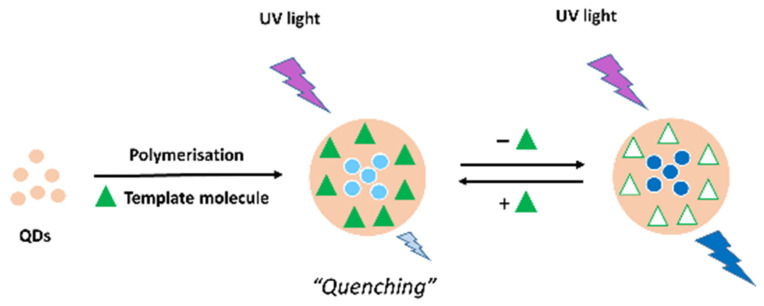
Schematic illustration of the general preparation procedure and possible sensing mechanism of QD-MIPs.

MIP-QDs sensors produce a measurable optical change during the specific rebinding to the target analyte. Figure 1 shows the schematic illustration of a general preparation procedure and a possible sensing mechanism of QD-MIP probes, where the intensity of fluorescence quenching is used for quantification. Accordingly, the excellent selectivity of MIPs combined with the high sensitivity of QDs-based fluorescence sensors could reduce the limits of detection (LODs) allowing the determination of target analytes at trace levels [8].

This review does not pretend to report an exhaustive collection of MIP-QDs-related papers but to provide a general overview of the different strategies for the preparation of MIP-QDs optical sensors, with emphasis on the modifications that represent an improvement in the sensor performance. The synthetic strategies are divided into three categories according to their chemical composition which includes silica-based MIP-QDs, hybrid inorganic-organic MIP-QDs, and organic MIP-QDs sensors. Finally, recent applications of MIP-QDs in sensing and bioimaging are presented.

## 2. QDs Used in Combination with MIPs in Sensor Preparation

QDs are semiconductor nanoparticles that have unique electronic and optical properties due to the quantum effects that appear in nanoscale particles. For this reason, QDs have been used for the preparation of nanosensors as they provide advantages respect to conventional probes based on organic or inorganic fluorescent dyes. Some of the interesting optical properties include high fluorescence quantum yield (QY), broad excitation, narrow emission band and good photostability. Moreover, the emission wavelength of QDs can be tuned by controlling their chemical composition and particle size. Therefore, the development of QDs-based sensors has rapidly increased due to their simplicity, fastness and high sensitivity.

QDs can be divided into two groups according to their composition: inorganic QDs, which can be found in different configurations including binary, ternary, and core-shell structures, and organic QDs which are graphitic structural materials like graphene quantum dots (GQDs) and carbon dots (CDs) [9].

In particular, inorganic QDs, commonly used in the synthesis of MIP-QDs sensors, are among binary semiconductors nanocrystals composed of elements from groups II–VI, III–V and IV–VI of the periodic table. Initial works were based on CdSe and CdTe QDs [10,11,12,13], but the toxicity of cadmium has led to progressive use of zinc such as ZnS and ZnSe QDs [14,15]. Besides, it is common to use metals such as Mn and Cu for the preparation of doped ZnS QDs since they provide interesting advantages including lower self-quenching, and superior resistance to thermal, chemical and photochemical disturbances [14,16,17,18]. Regarding core-shell structures, the presence of a shell can improve the quantum yield (QY) of the core and, at the same time, protect the core, thus increasing its photostability. ZnS QDs are frequently used as the shell while the core is composed by elements from groups II–VI, III–V and IV–VI such as CdSe/ZnS QDs and CdTe/CdS QDs [19,20].

Although organic QDs are not the topic of the present review, relevant examples of MIP-QDs sensors prepared with GQDs and CDs can be found in literature and are also covered in the present review [21,22].

Several kinds of QDs are currently available on the market, however they are usually synthesized in the laboratory. Regardless their origin, the QDs usually have to be modified in order to keep them kinetically stabilized and to obtain a more suitable surface chemistry for the subsequent surface modification protocols. For example, the introduction of an optically transparent silica coating serves as a protective shield to the QDs and also provides silicon hydroxyl groups on the surface, which are needed in the subsequent introduction of functional monomers during the imprinting process. The most common paths for coating silica shell on QDs are sol-gel reaction of tetraethyl orthosilicate (TEOS), and/or [3-(methacryloxy)propyl]trimethoxysilane (MPTS) [23,24]. Besides, modifications with thioglycolic acid (TGA) providing hydrophilic characteristics to QDs have been frequently described [20,25,26,27]. Likewise, modifications with L-cysteine (L-Cys) provides water compatibility, improving stability and dispersion of QDs, as well as important binding groups for molecularly imprinting such as –NH_2_ and –COOH [28].

## 3. Strategies for the Preparation of MIP-QDs Optical Sensors

### 3.1. Silica-Based MIP QDs Sensors

The use of silica for the modification of QDs sensors has received considerable attention in the last years due to the numerous benefits that silica brings to these composites. In this sense, silica is transparent in the UV-Vis spectrum, protects QDs against photo-chemical degradation and preserves the optical properties of QDs. Moreover, the silica coating impedes the diffusion of heavy metal ions into the surrounding environment and also provides hydrophilic properties, biocompatibility and the possibility of surface functionalization. Additionally, silica has a highly cross-linked rigid matrix that is ideally suitable for the formation of recognition sites [9,10]. The sol-gel method is a simple and relative low-cost technique to prepare different sorts of nanomaterials or to modify polymer surfaces. Particularly, MIPs prepared by this way present selective cavities with long lifetime because silica-based materials are strong and stable structures with high thermal and mechanical stability. Moreover, the removal of the template molecule from the polymer is more efficient in MIPs prepared by sol-gel methodology [29].

There are two principal methods for the preparation of MIP-QDs sensors by sol-gel method: the Stöber method and the reverse phase microemulsion method. The preparation of silica-based MIP QDs by the Stöber method has been widely described in scientific literature. The QDs used in the protocols should be modified before the imprinting process. Examples include TGA-capped CdTe@CdS [20], MPTS-Capped Mn-Doped ZnS QDs [23,30], CdTe@SiO_2_ QDs [31], TGA-capped CdTe QDs [25,26], SiCQDs [32], and APTS-modified GQDs [21]. In general, the polymerization protocol described for Stöber method includes a polymerization mixture formed by (3-aminopropyl)triethoxysilane (APTES) as functional monomer, TEOS as cross-linker, NH_3_·H_2_O as a catalyst and the chosen template. First, APTES is frequently incubated with the template to form a pre-polymer through hydrogen bonding between the amino groups (−NH_2_) in the molecules of APTES and the functional groups in the template molecules. After a short period of incubation, TEOS is added and the pre-polymer is immobilized on the surface of the QDs by silanization reaction through the simultaneous hydrolytic condensation of APTES with TEOS using NH_3_·H_2_O as the sol-gel catalyst. After polymerization, the MIP-QDs are collected by centrifugation, and then washed to remove the template molecules. As a control material, non-imprinted polymers (NIPs) are synthesized in the same way except for the addition of the template. Silica-based MIP-QDs sensors prepared in this manner have been successfully applied for the optosensing determination of perfluorooctanoic acid [20], pentachlorophenol [23], amoxicillin [26], patulin [30], p-aminophenol [31], indoxacarb [32], metronidazole [21] and sinapic acid [33] in different matrices being water, food and biological samples the most common. The reported imprinting factors (IFs) ranged from 2.0 to 4.4 with the exception of the MIP-QDs sensor for the detection of amoxicillin in egg, milk and honey samples which reached the value of 43 [26]. The LODs obtained ranged from 0.14 µg L^−1^ to 25.7 µg L^−1^.In general, the resulting developed sensors showed good sensitivity and accuracy with great potential as luminescent nanosensors in analytical applications.

An interesting modification to produce a mesoporous structure on the MIP-QDs consisted in the addition of a structure-directing surfactant, as cetyltrimethylammonium bromide (CTAB), during the one-pot synthesis. CTAB helped in the formation of a mesoporous structure extending the surface of the silica imprinted polymer, thus improving diffusion of target analytes [34]. For example, Jalili et al. [8] obtained a specific surface area of MIP-QDs prepared in the presence of CTAB 40 times higher than the corresponding MIP-QDs prepared without CTAB, thus confirming the mesoporous structure.

In parallel, there was a need to develop strategies that allowed the use of hydrophobic QDs, which have excellent fluorescent properties, in hydrophilic samples. Water-in-oil (W/O) reverse microemulsion has been the followed strategy leading to QDs with improved dispersibility in aqueous solution, and an increased fluorescence intensity [19,35]. The incorporation of hydrophobic QDs in silica spheres using a reverse microemulsion method was reported for the first time by Koole et al. in 2008 [36]. Since then, the reverse microemulsion method, with slight modifications, has been frequently used for the preparation of silica-based MIP-QDs sensors [9]. Generally, in the protocols described for the MIP-QDs sensors preparation by reverse microemulsion method, the used QDs were usually modified before the imprinting process. Some examples are 3-mercaptopropionic acid (MPA)-capped CdTe QDs [13], trioctylphosphine oxide (TOPO)-modified CdSe/ZnS QDs [19], TGA-capped CdTe QDs [12,37], CdS/CdSe/ZnS QDs [38] and a novel ecofriendly FeSe-QDs [1]. Turning to the protocols for the preparation of MIP-QDs sensors by this method, cyclohexane was used as the continuous phase, whereas TritonX-100 and n-hexanol were commonly used as surfactant and co-surfactant, respectively. Experimentally, the aqueous solution of QDs and ammonia are emulsified under magnetic stirring forming water micro-droplets. Then, TEOS is introduced and easily dissolved in cyclohexane, which pass the cyclohexane/water interface by diffusion, starting to hydrolyze upon the catalysis of ammonia. The hydrolysis produces a deprotonation of the silanol groups making the partly hydrolyzed TEOS molecules to be hydrophilic enough to enter into the aqueous phase. Then, the functional monomer APTES and the template are added and the MIP is synthesized via hydrolysis and condensation reactions of APTES and TEOS in the presence of the template. APTES not only covers the QDs, but also provides surface amino groups (-NH_2_) that interact with the template molecules via hydrogen bonds forming recognition sites in the surface of the silica matrix. The microemulsion is broken by acetone, subjected to centrifugation, and the resultant precipitate is sequentially washed with ethanol and water. Finally, template is removed from the silica matrix with an appropriate solvent. Silica-based MIP-QDs sensors prepared by modified reverse microemulsion method has been successfully applied for the determination of malachite green [13], N^e^-carboxymethyllysine [19], chloramphenicol [37], prilocaine [12], saxitoxin [39], cyfluthrin [1] and cypermethrin [38] in different types of samples such as food, biological and environmental samples. The LODs obtained were as low as 0.132 µg L^−1^ and 0.3 µg kg^−1^.

### 3.2. Hybrid Inorganic-Organic MIP-QDs Sensors

Despite the mentioned advantages of the use of silica for coating QDs, there are also drawbacks such as the limited porosity of the imprinted layer and the scarce modifiable chemical functionalities. However, organic polymers possess a larger choice of functional groups due to the wide functional monomers and cross-linkers that are commercially available. Thus, the hybrid inorganic-organic MIP-QDs sensors could combine the benefits that silica gives to the optical properties of the QDs, while the recognition of target molecules is performed by the versatile organic imprinted polymer.

The protocols for the synthesis of hybrid inorganic-organic MIP-QDs sensors described in literature are quite different in terms of configuration, where QDs may act as sensing material and/or as supporting material remaining in the core of the final composite. In the latter case, silica works as a bridge between the QDs and the organic polymer. For instance, a fluorescent nanosensor for the optosensing determination of cyfluthrin was prepared using FeSe-QDs as sensing material but also as a support material. The synthesis of the composite was performed by a modified reverse microemulsion method. Firstly, a solution composed of 2, 2′-azobisisobutyronitrile (AIBN), TritonX-100, cyclohexane, TEOS, FeSe-QDs and ammonia was used and the hydrolysis of TEOS performed. Then, an already prepared pre-polymerization mixture containing the template, the monomer APTES, the secondary monomer methacrylic acid (MAA) and the cross-linker ethyl glycol dimethacrylate (EGDMA) was mixed to the previous one and subsequently heated to start the polymerization. The MIP-FeSe-QDs sensor was applied for the optosensing determination of cyfluthrin in fish and sediment samples obtaining satisfactory recoveries and LODs of 1.0 µg kg^−1^ and 1.3 µg kg^−1^ in sediment and fish samples, respectively [1]. In a similar manner, a fluorescence probe for the detection of cyphenothrin in river water was prepared. Briefly, Mn-doped ZnS QDs were synthesized and stabilized with a silica coating by hydrolysis of TEOS. Then, APTES was connected to the silica by silanization providing amino groups to the surface of the obtained material. Finally, a MIPs shell was synthesized by surface imprinting on the amino modified QDs through self-assembling of the functional monomer acrylamide (AM), the cross-linking agent (EGDMA) and the template (cyphenothrin). The resulting fluorescent probe showed a LOD of 9.0 nmol L^−1^ and performance characteristics comparable or superior to other methods for the analysis of cyphenothrin reported in literature [40].

**Figure 2:**
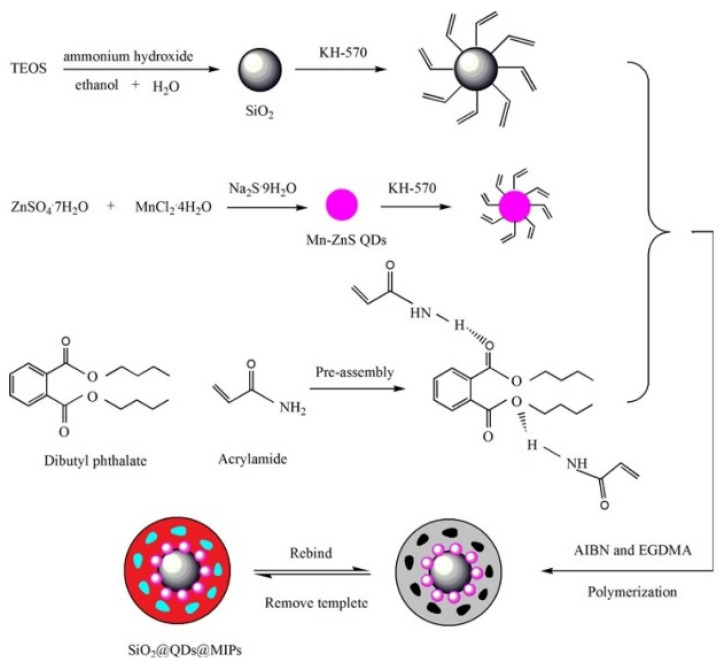
Schematic preparation of a fluorescent hybrid inorganic-organic MIP-QD sensor for the determination of dibutyl phthalate (DBP). Reproduced with permission from [17]. Copyright 2017 Elsevier.

However, there are some drawbacks related to the incorporation of QDs into silica spheres in terms of fluorescence intensity and morphology of MIPs. For this reason, a different approach has been proposed in which QDs are located outside the core of the composites. In this case, silica nanoparticles are proposed as support materials where surface MIPs layers can be anchored, and QDs are placed on the surface or in its proximity, thus enhancing the fluorescence intensity. At the same time, the imprinted cavities remain also on the surface, improving mass transfer, recognition ability and separation efficiency. Experimentally, the support and sensing materials are synthesized and modified separately and then added to the polymerization mixture. A remarkable example of this configuration is the preparation of a fluorescent molecularly imprinted (SiO_2_@QDs@MIPs) sensor for the determination of dibutyl phthalate (DBP) and the experimental procedure is depicted in Figure 2. Briefly, vinyl-modified SiO_2_ nanoparticles were used as core substrate and vinyl-modified Mn-doped ZnS QDs nanoparticles were used as the sensing material. Then, the MIP was synthesized by precipitation polymerization on the surface of vinyl-modified SiO_2_ nanoparticles and the Mn-doped ZnS QDs become embedded into the MIP. The developed SiO_2_@QDs@MIPs showed a rapid response for the fluorescent detection of DBP, reaching a LOD of 1.13 µg L^−1^, thus representing a clear alternative analytical tool for the analysis of DBP [17].

In a similar manner, MIPs/QDs@SiO_2_ fluorescence sensor via a custom-tailored strategy for the determination of sulfonamides (SAs) in river water was described [41]. First, SiO_2_ particles were synthesized by the Stöber method and subsequently modified with TEOS and acryloyl chloride to form the AM-APTES-SiO_2_, which worked as support material. Secondly, CdTe QDs were synthesized and modified by the polymerizable surfactant octadecyl-4-vinylbenzyldimethylammonium chloride (OVDAC), to form OVDAC/CdTe QDs that worked as the fluorescent material. Finally, the prepared materials were added to the polymerization mixture together with SAs, AM and EGDMA to carry out a two-step imprinting polymerization process in acetonitrile. During the polymerization process, the modified spherical SiO_2_ served as support material but also formed hydrogen bonds with SAs and, in addition, π–π interaction might be formed by OVDAC and SAs. The MIP layers were formed on the surface of the SiO_2_ particles with the QDs embedded in the polymer shell. The LOD obtained was of 29.27 µg L^−1^ and a relatively high IF of 6.63 was obtained probably due to the presence of multiple interactions provided both by silica and OVDAC.

### 3.3. Organic MIP-QDs Sensors

Despite the fact preparation of MIP-QDs has been mainly based on the use of silica, organic imprinted polymers have emerged as an interesting alternative. In this sense, a comparative study of the synthesis of tetracycline-imprinted polymeric silicate and acrylate on CdTe quantum dots as fluorescent sensors showed that acrylate-based MIP presented better specificity and selectivity than silica-based MIP, as well as, fewer non-specific interactions. Thus, a better fluorescence quenching of the QDs should be expected for acrylate-based MIPs on the basis of the stronger interaction that occurs between the template and the acrylate-based polymer. This would involve an increase in the sensitivity for the acrylate-based MIP sensors [42].

**Figure 3:**
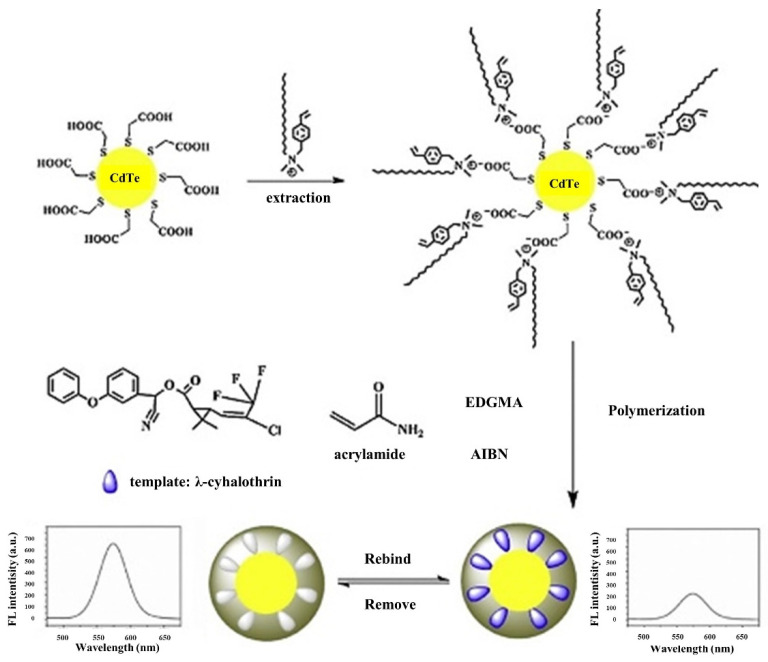
Schematic illustration for the preparation of MIPs-OVDAC/CdTe QDs. Reproduced with permission from [44]. Copyright 2016 Elsevier.

Despite the use of organic polymers for the preparation of MIP-QDs sensors is a promising combination, QDs combined with organic MIPs have been less reported. MIPs based on organic polymers are mainly synthesized by chain-growth polymerization of cross-linker and functional monomer with radical initiator. However, before the imprinting, QDs must be functionalized with an anchor such as TGA or 4-vinylpyridine that was used in the first organic-based MIP-QDs preparation works [43]. Alternatively, as depicted in Figure 3, Wei et al. [44] proposed the use of OVDAC to form polymerizable vinyl groups on CdTe QDs for the subsequent polymerization and also to assist the transference of aqueous CdTe QDs to an organic solvent. Thus, the modified vinyl end layers could direct the imprinting polymerization selectively occurring at the surface of CdTe QDs. In this particular case, OVDAC/CdTe QDs were simultaneously used as solid support, optical material and auxiliary monomer. Then, a surface imprinting-directing precipitation polymerization was carried out in two steps. By this manner, covalent bonds were formed by copolymerizing AM and EDGMA with OVDAC via its p-vinylbenzyl groups present on the CdTe surface in the presence of λ-cyhalothrin as template molecule. The prepared MIPs-OVDAC/CdTe QDs showed higher molecular selectivity and sensing specificity compared with two types of molecularly imprinted silica coated QDs prepared by the Stöber method and the reverse microemulsion method. The MIPs-OVDAC/CdTe QDs sensor showed an IF of 5.99 and provided a LOD of 13.49 µg L^−1^. The proposed fluorescent sensor was successfully applied for the determination of λ-cyhalothrin in river water samples. This synthesis approach represents a general strategy for the preparation of high-performance fluorescent sensors based on QDs.

Furthermore, efforts have been made to reduce the number of preparation steps and to shorten the preparation time. For example, ultrasound irradiation was a novelty used for the synthesis of polyethylenglycol-Mn-doped ZnS QDs as well as for the preparation of the MIP-QDs composites by precipitation polymerization. The MIP was synthesized using cocaine (COC) as template, ethylene dimethacrylate (EDMA) as the functional monomer, divinylbenzene (DVB) as the cross-linker, and AIBN as the initiator. Polymerization was carried out by ultrasound irradiation at room temperature for 4 h. This sensor showed recognition of COC and its metabolites benzoylecgonine and ecgonine methyl ester, presenting an IF values of 23, 7.9 and 9.1, respectively, whereas fluorescence quenching was not observed for other drugs of abuse and metabolites [14]. In a similar manner, a MIP- Mn-doped ZnS QDs fluorescent probe for the screening of aflatoxins (AFs) in non-dairy beverages was described. The developed fluorescence probe was found to be selective for four AFs (AFB1, AFB2, AFG1, and AFG2) and provided a LOD of 16 µg L^−1^ which is near to the maximum total AFs levels permitted in foodstuffs by European authorities [45].

**Figure 4:**
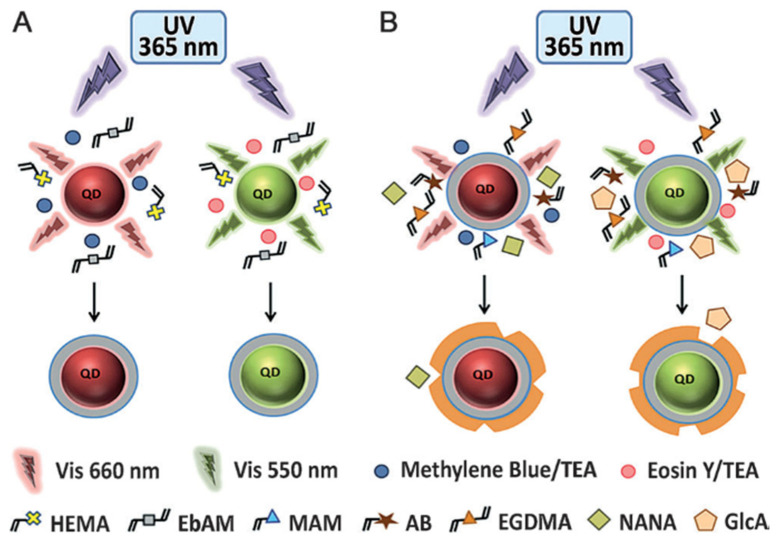
(**A**) Red or green light emitted from InP/ZnS QDs excited by UV irradiation is used to synthesize a polymeric shell in situ around the particles by photopolymerization. Methylene blue/trimethylamine (TEA) are used as the initiator system for red-QDs and eosin Y/TEA for green-QDs; (**B**) A second shell of MIP is synthesized by reinitiation in the presence of functional and cross-linking monomers and a molecular template (GlcA or NANA). Reproduced with permission from [46]. Copyright 2016 John Wiley & Sons.

In addition, a new strategy was proposed for grafting MIPs on QDs by means of physical entrapment, thus, eliminating the need of chemical modification of the QD surface or chemical coupling. Panagiotopoulou et al. [46], described the preparation of fluorescent core-shell particles specifically recognizing glucuronic acid (GlcA) or N-acetylneuraminic acid (NANA) using the emission of the QDs as an internal light source for photopolymerization. Simultaneous multiplexed labeling of human keratinocytes with green QDs conjugated with MIP-GlcA and red QDs conjugated with MIP-NANA was demonstrated by fluorescence imaging. In this work, as schematically depicted in Figure 4, QDs were excited with an UV lamp (365 nm) and the emitted light was used by the initiator, which had an absorption wavelength that overlaps the emission of the QDs. The weak emitted light loses intensity from the QDs surface to the bulk phase due to absorption of the initiators. Thus, the photopolymerization occurred near the QDs surface forming a thin film whose thickness is controlled by the radiation time.

GQDs have also been used as fluorescence material in the preparation of nanosensors combined with MIP of organic nature, offering an eco-friendly alternative to avoid the use of QDs that contain toxic metals. In this sense, a MIP-GQDs fluorescent nanosensor for detection of methamphetamine was proposed. Briefly, triethoxyvinylsilane was used to functionalize reduced GQDs with vinyl groups, and the MIP was synthesized using MAA as functional monomer, EGDMA as cross-linker, AIBN as initiator and methamphetamine (METH) as template. The mixture was stirred and placed in a water bath at 60 °C for 3 h. The sensor showed a LOD of 1.7 μg L^−1^, a good selectivity for METH and did not show response to other analytes such as amphetamine, ibuprofen, codeine, and morphine [47].

## 4. Selected Applications

### 4.1. MIP-QDs Optical Sensors

MIP-QDs optical sensors are able to produce a measureable optical change during a binding event. Particularly, when target species bind the imprinted cavities that are present in the MIP-QDs composites, the fluorescence emitted by the sensing material suffers a change that can be easily measured. In addition, fluorescence sensors have attracted considerable attention as a consequence of the low LODs that can be achieved coupled with the simplicity of the format. Moreover, traditional analytical methods require high consumption of reagents and time, costly equipment and complicated sample preparation. On the contrary, methods based on MIP-QDs optical sensor are easy, fast, sensitive and versatile allowing the determination of a wide range of analytes including non-fluorescent compounds in a variety of complex matrices [9,48]. The MIP-QDs optical sensors described in scientific literature may be categorized in two groups: solution-based probes and solid materials-based probes.

#### 4.1.1. Solution-Based Probes

Most of the sensing systems described in literature are among this category. Briefly, a fixed concentration solution of MIP-QDs composites is prepared in a proper medium, usually a buffer. Then, known concentrations of analyte standard solutions are sequentially added and stirred. After a fixed period of time, the luminescence signal can be measured with an appropriate equipment such as a spectrofluorometer. Parameters including pH, temperature, incubation time, and working concentration of MIP-QDs composites should be optimized. In short, the fluorescence is measured in the absence and presence of the target analyte and recorded as F_0_ and F, respectively. The value of F_0_-F is calculated as a response function to evaluate the analyte concentration. Consequently, measurements can be performed in a few minutes, with low consumption of time and reagents. Hence, solution-based MIP-QDs sensors have been, and still are, the most reported for the determination of several target analytes in a great variety of samples. For example, in the environmental area, cyfluthrin [1], sulfonamides [41], dibutyl phthalate [17], and perfluorooctanoic acid [20] have been determined mainly in water samples. Within the field of food security, patulin [30], aflatoxins [45], saxitoxin [39] and malachite green [13] have been studied in foodstuffs. In the biochemical field, pharmaceuticals and drugs of abuse such us prilocaine [12], promethazine hydrochloride [49], metronidazole [21], p-aminophenol [31], methamphetamine [47] and dopamine [50] have been determined in biological samples such as plasma and urine.

Recently, an eco-friendly fluorescence nanosensor based on MIP-coated silica-carbon quantum dots (MIP@SiCQDs) for the detection of indoxacarb (IXC) has been developed. In this work, SiCQDs were synthesized in one step and then the MIP nanocomposite was prepared by a sol-gel method anchoring the MIP matrix on the SiCQD surface. The analytical performance was optimized and revealed a linear correlation between the fluorescence quenching and the increase of IXC concentration within the concentration range of 4–102 nM, with a LOD of 1 nM (0.53 µg L^−1^), being comparable or better to other MIP-QDs sensor described in literature. The practical application of the proposed sensor was evaluated for the detection of IXC in well water, fruit and tomato samples obtaining recoveries between 95 and 106% with a relative standard deviation below 6.0%. Thus, the utility of the proposed sensor for the measurement of IXC in real samples was demonstrated. Besides the advantages of this approach, such as low cost and facile preparation, the sensor could be reused without any appreciable loss of fluorescence quenching at least ten times following an adequate washing procedure [32].

In another work, a MIP-CdTe QDs photoluminescence sensor was developed for the determination of amoxicillin in food samples. In short, TGA-capped CdTe QDs were first synthesized and subsequently embedded in a silica-based MIP by a sol-gel process. After proper optimization of experimental variables, an IF of 43.6 was obtained demonstrating the presence of specific cavities in the MIP-CdTe QDs. The proposed sensor showed a high response to amoxicillin whereas sensor response to other antibiotics, such as ampicillin, cephalexin, penicillin G, chloramphenicol, and thiamphenicol, was much lower confirming the high selectivity of the proposed sensor. Regarding the analytical performance, the prepared sensor presented a linear photoluminescence quenching for amoxicillin detection in the concentration range from 0.20 to 50.0 µg L^−1^ and a LOD of 0.14 µg L^−1^. The LOD obtained was much lower than other methods previously published for amoxicillin detection. Finally, the proposed sensor was applied for the determination of amoxicillin in egg, milk and honey samples and recoveries obtained for spiked samples were in the range of 85.3–102.0% with a RSD below 6%, confirming the usefulness of the proposed device for the highly sensitive and selective determination of amoxicillin [26].

Despite all the advantages and conveniences solution-based probes can provide, there are still some serious limitations. The solution sensor is frequently used only once before being disposed, which can raise the cost for their routine application and can lead to environmental contamination. Furthermore, sensor solutions may degrade with time becoming unstable, inhibiting their use as portable sensor devices.

#### 4.1.2. Solid Materials-Based Probes

The incorporation of QD sensors into solid materials has been proposed as an alternative solution to the drawbacks presented in solution-based probes. Particularly, MIP-QDs sensor microdevices (e.g., microfluidic system, test strips) can improve QD sensing applications due to their small size, simplicity of fabrication, portability and low sample volume, which is promising for performing reliable point-of-care testing and in-field diagnosing within a few minutes [51].

**Figure 5:**
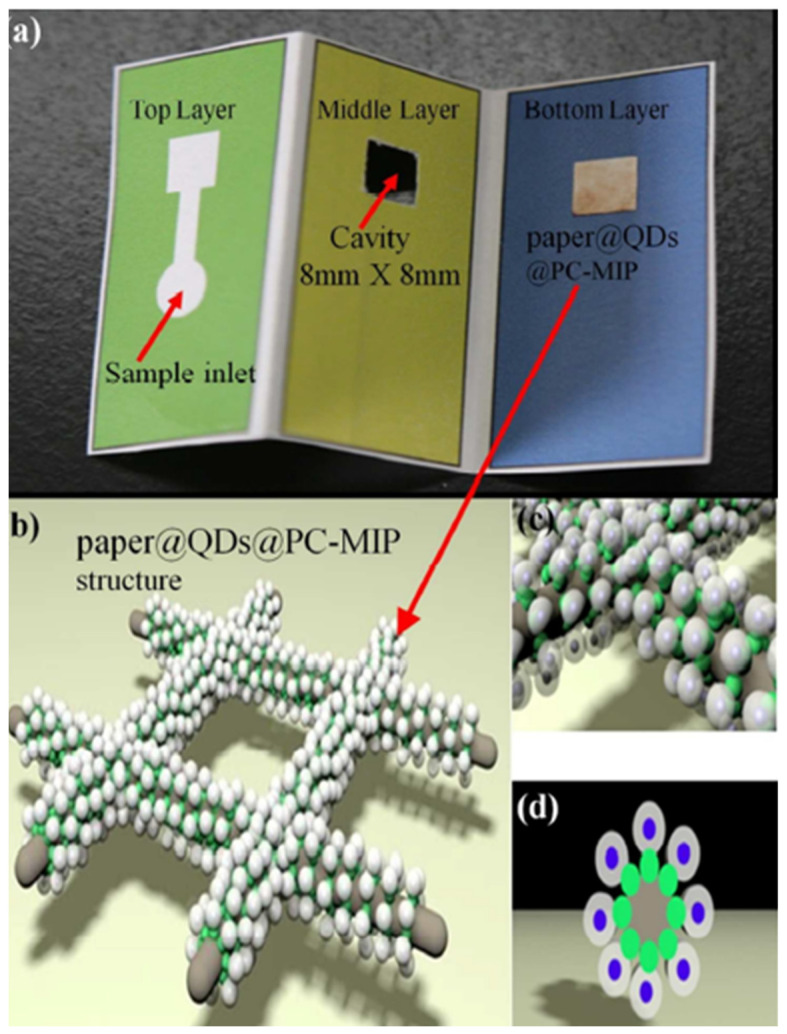
(**a**) Photograph of the paper@QDs@PC-MIPs µPADs; (**b**) Schematic illustration of the structure of the paper@QDs@PC-MIPs component; (**c**,**d**) Side and bottom view of enlarged diagram of the structure of the paper@QDs@PC-MIPs on cellulose paper. Green shell: fluorescent quantum dots; white shell: PC imprinting; gray column: cellulose paper. Reproduced with permission from [25]. Copyright 2017 American Chemical Society.

As an illustration, in a novel strategy, MIP-QDs were prepared on three-dimensional origami paper microfluidic chips for the fluorescence detection of phycocyanin [25]. In this work, a filter paper was used as support for the preparation of the microfluidic paper-based analytical device (µPAD) and it is schematically shown in Figure 5. Firstly, pieces of filter paper were functionalized with APTEs and then, TGA-capped CdTe QDs were grafted on the filter paper substrate to form the paper@QDs. Finally, the paper@QDs@PC-MIPs were prepared by surface imprinting. The chip was designed and subsequently printed in a filter paper with the help of a wax printer to form a hydrophobic barrier. In brief, when the phycocyanin sample flowed through the channel and reached the paper@QDs@PC-MIP, the quenching of QDs fluorescence could be recorded. A lamp set through the solid sample holder excited the paper@QDs@PC-MIPs µPADs and the emitted fluorescence was recorded using a spectrofluorometer. A dynamic range from 10 to 50 mg L^−1^ was obtained and with a LOD of 2 mg L^−1^. The proposed µPADs showed higher selectivity towards phycocyanin than towards other related compounds. The application in lake water samples and seawater samples showed recoveries higher than 94.3% with RSDs below 5.7%, demonstrating that the proposed µPADs could be used for the determination of phycocyanin in environmental water samples. Additionally, the sensor presented numerous advantages such as miniaturization, stability for at least 7 days, portability, and the possibility of reuse the sensor after a proper analyte elution.

Recently, another ZnSe QDs-based MIP paper microfluidic device was developed with some design modifications that permitted the multiplexed detection of cadmium (Cd^2+^) and lead (Pb^2+^) ions. The multiplexed detection was achieved thanks to the use of a rotary μPAD. The developed sensor presented a linear response from 1 to 70 µg L^−1^ with a LOD of 0.245 µg L^−1^ for Cd^2+^ µPAD while the Pb^2+^ µPAD showed a linear response from 1 to 60 µg L^−1^ with a LOD of 0.335 μg L^−1^. Recoveries obtained for spiked sea and lake water samples were from 95% to 105% with the RSDs below 5.6%. Both LODs obtained were below the maximum residue levels (MRL) for drinking waters set by the United States Environmental Protection Agency and thus appropriate for the detection of Cd^2+^ and Pb^2+^ in water [15].

### 4.2. MIP-QDs as Ratiometric Fluorescence-Based Sensors

Most of the reported MIP-QDs in scientific literature are single wavelength intensity modulation type sensors. These probes usually present some common drawbacks such as nonhomogeneous distribution, undesirable variations in the concentration of the sensor and also can be affected by the environmental conditions [34]. For this reason, the ratiometric fluorescent determination has emerged as an interesting alternative strategy for the detection of target analytes. The probe is usually composed of two emitting materials: the sensitive material whose fluorescence can be quenched in the presence of the target analyte, and the reference material whose fluorescence remains constant. Thus, the use of the ratio between the two fluorescence signals well resolved, circumvents the common drawbacks of single wavelength systems providing self-calibration, excellent selectivity and fast monitoring [52]. Due to the already mentioned intrinsic self-calibration, environmental disturbances can be avoided providing accuracy and higher sensitivity to trace analytes. Accordingly, in the last few years an increasing number of molecularly imprinted ratiometric fluorescence nanosensors have been proposed for the determination of various target analytes, such as malachite green in river and lake waters [34], folic acid in serum and food [53,54], sinapic acid in seeds [33], and dopamine [55] among others.

**Figure 6:**
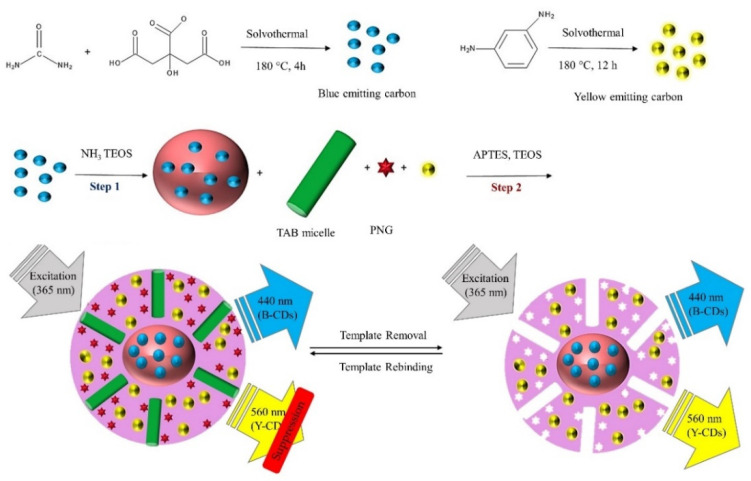
Schematic illustration of the fabrication process B/YCDs@mMIPs ratiometric sensor and the sensing mechanism of PNG. Reproduced with permission from [8]. Copyright 2020 Elsevier.

As an illustrative example, depicted in Figure 6, Jalili et al. prepared a ratiometric fluorescent sensor using blue (B) and yellow (Y) emitting CDs (B/YCDs) in a mesoporous MIP (B/YCDs@mMIP) for the detection of penicillin-G (PNG) in milk. During the rebinding of PNG, the fluorescence of yellow emissive CDs was quenched while the reference blue emissive CDs remained constant, resulting in a variation of the fluorescence color from yellow to blue. The composite was prepared by a one-pot sol-gel based method in two steps in the presence of the structure-directing surfactant CTAB. In the first step, the B-CDs were incorporated into silica-spheres forming the B-CDs@SiO_2_. In the second step, a Y-CDs embedded mesoporous imprinted polymer layer was formed on the surface of the B-CDs@SiO_2_. The sensor showed a linear response to concentrations of PNG in the range of 1–32 nM with a LOD of 0.34 nM (0.113 µg L^−1^). Recognition sites were formed in MIP sensor showing an IF of 5.2. The higher specific surface provided by the mesoporous structure enhanced the sensitivity and shortened the response time. The proposed sensor showed good selectivity to PNG over some structural analogues such as nafcillin, dicloxacillin and oxacillin. Moreover, the effect of interfering substances commonly found in milk samples caused a relative error of less than 5%, which was within the tolerable values and therefore showed high selectivity. The analysis of spiked real milk samples provided recoveries ranging from 98% to 103% with RSD values below 0.9% which demonstrated the potential of the sensor for a simple, fast, eco-friendly, sensitive and selective detection of PGN in milk samples [8].

Signal variation of the ratiometric fluorescent probe can be observed by the naked eye which makes the sensor suitable for semi-quantitative determinations in medical fields such as point-of-care testing and daily monitoring [27,33,53,54]. This was nicely demonstrated by Wang et al. in the molecular imprinting based hybrid ratiometric fluorescence sensor for visual determination of bovine hemoglobin (BHb) [56]. Firstly, green CdTe@MIPs and red CdTe@SiO_2_ were synthesized separately and subsequently mixed according to an appropriate proportion to obtain the ratiometric fluorescent system. The fluorescence emitted by the green CdTe@MIPs was quenched due to the rebinding of BHb at the imprinted polymer recognition sites, while the red fluorescence emitted by CdTe@SiO_2_ was not altered during the binding event. As a consequence, the variation of the fluorescence ratio resulted in a distinguishable color change from green to red. The optimal ratio for CdTe@MIPs and CdTe@SiO_2_ in the ratiometric sensor was optimized to obtain a discernible wide color change from light green to orange red and to red. The linear relationship between the ratio of the fluorescence intensities and the BHb concentration was in the range of 0.050–3.0 µM with a LOD of 9.6 nM. Good recognition specificity for BHb over other proteins was observed allowing its determination in urine samples with quantitative recoveries (95.7–101.5%, RSDs below 4.2%).

**Figure 7:**
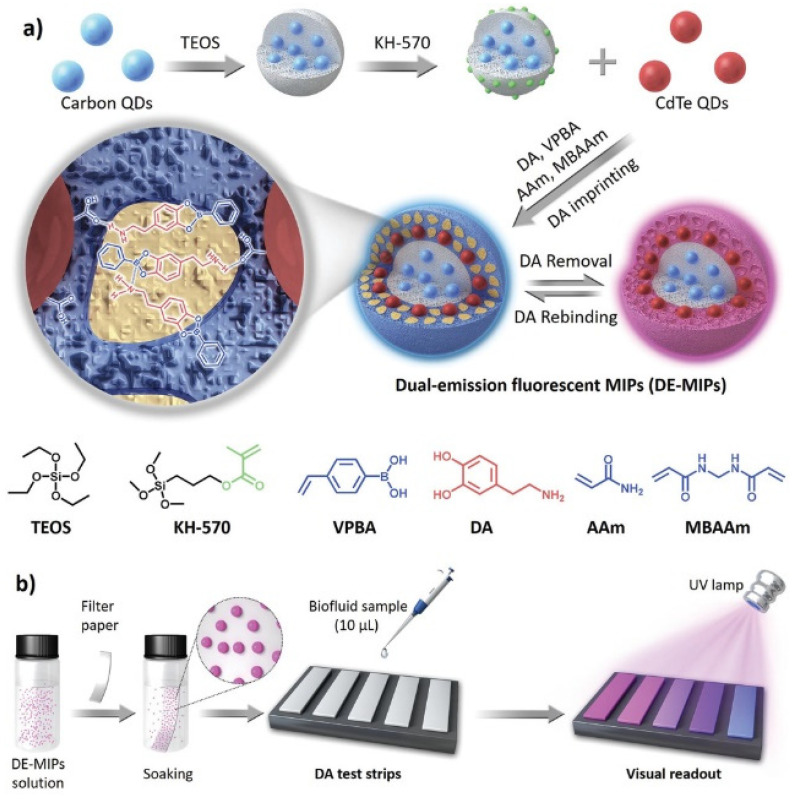
(**a**) Schematic generation of dual-emission fluorescent molecularly imprinted polymer nanoparticles (DE-MIPs) with specific DA affinity; (**b**) The DE-MIPs-coated filter paper as a facile DA test strip for visual detection. Reproduced with permission from [27]. Copyright 2019 John Wiley & Sons.

Ratiometric detection can also be performed in form of test strips prepared by grafting the MIP-QDs sensors on paper surface. At this regard, Wang et al. [27], developed molecularly imprinted fluorescent test strips for the direct visual detection of dopamine in biofluids, as depicted in Figure 7. Blue-carbon QDs were embedded in silica by precipitation of TEOS and worked as the constant reference signal. Then, the MIP was prepared by surface imprinting on the surface of the prepared silica nanoparticles using red-CdTe QDs as variable fluorescence signal, AM as the backbone monomer, 4-vinylphenylboronic acid (VPBA) as functional monomer, and methylene bisacrylamide (MBAAm) as cross-linker in the presence of dopamine. Then, pieces of filter paper tailored to 4 × 10 mm were soaked in a solution of MIP-QDs in ethanol, taken out and dried to get the test strips. The analysis of real serum samples demonstrated the semi-quantitative visual detection of dopamine in serum providing detection limits of (100–150) × 10^−9^ M by the naked eye. Moreover, the visual readout was achieved in 3 min using 10 µL of sample. Thus, the proposed test strips could be applied to facile and fast daily monitoring of dopamine in serum samples without expensive instruments.

### 4.3. MIP-QDs in Bioimaging

QDs have been widely used for bioimaging and labeling probes and their combination with MIPs extends the range of application for optical sensing, imaging diagnostics, and controlled drug release. In fact, MIP-QDs have demonstrated some advantages over antibodies such as high stability and long shelf life, lower cost of preparation, shorter preparation time and easier functionalization. Additionally, MIP-QDs have shown lack of immunogenic response, high affinity and specificity, good biocompatibility, solubility and ability to cross the cell membrane. For this reason, in the last years MIP-QDs have been seen as a powerful tool for imaging of tumor biomarkers. As a remarkable example, Cecchini et al. demonstrated that MIP-QDs may represent a possible alternative to conventional monoclonal antibodies. In this work, a MIP-QDs sensor to detect human vascular endothelial growth factor (hVEGF), which plays a crucial role in tumor progression, was prepared. The emitting CdTe QDs were covalently coupled onto the MIP surface after the polymerization process. In essence, the proposed MIP-QDs nanoprobe was able to selectively bind to the entire protein in vitro, and to localize cancer cells overexpressing hVEGF in tumor xenograft in vivo experiments [57].

Later on, another MIP-carbon QDs composite was used as a biocompatible optical imaging device for probing cancer biomarkers. The MIP was prepared for the recognition of glucuronic acid (GlcA), which is a substructure of hyaluronan, a biomarker for certain cancers. A thin MIP shell was synthesized around N-doped carbon QDs, using the internal emission of the CQDs as a source for photopolymerization. Finally, the nanoprobe was able to distinguish effectively between tumor and healthy cells, thus being a useful tool for biotargeting and bioimaging of hyaluronan on fixated human cervical cancer cells [22].

**Figure 8:**
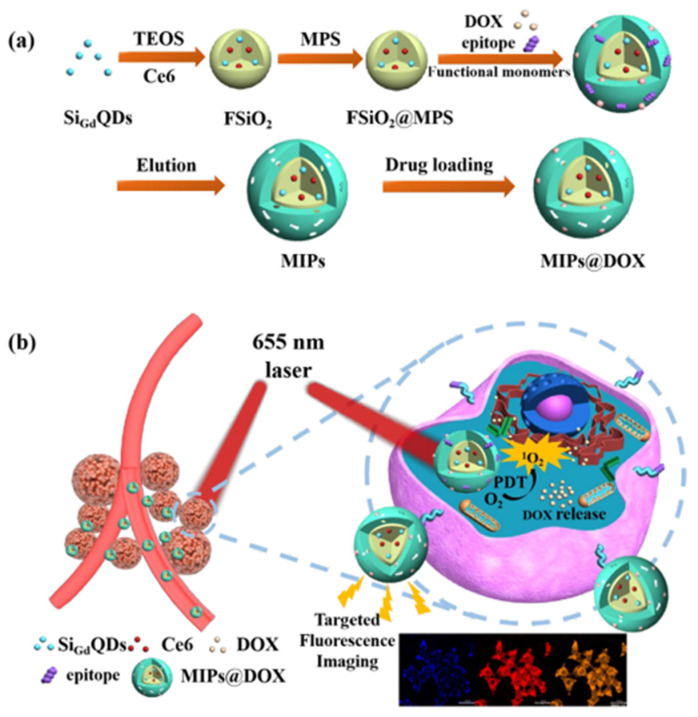
(**a**) Schematic illustration for the preparation of MIPs@DOX; (**b**) Schematic illustration of MIPs@DOX for targeted chemo-photodynamic synergistic treatment of tumor in vivo. Reproduced with permission from [58]. Copyright 2020 American Chemical Society.

More recently, Peng et al. [58] developed an interesting device to target cancer cells and efficiently improve the therapeutic efficacy of various therapies at the same time and it is illustrated in Figure 8. Gadolinium-doped silicon quantum dots and chlorin e6 were embedded in silica nanoparticles by a sol−gel method forming the fluorescent silica nanoparticles (FSiO_2_).Then, the imprinted layer was synthesized on the surface of FSiO_2_ via free-radical precipitation polymerization with two template molecules: one was the epitope of CD59 protein, which is a biomarker for several solid cancers, and the other one was an antitumor agent used in chemotherapy called doxorubicin (DOX). The embedded chlorin e6 is a common photosensitizer able to generate high levels of reactive oxygen species under laser irradiation to kill cancer cells. This property, combined with the MIP ability for recognizing the tumor cells and releasing the loaded DOX, provided a synergistic cancer therapy. The use of two different sensing materials (gadolinium and QDs) allowed dual-modality imaging capacity: fluorescence imaging and magnetic resonance imaging. In vitro and in vivo experiments confirmed an exceptional tumor-targeting capability and a slow-down in the growth of cancer cells thanks to the synergistic therapy with minimal adverse effects.

## 5. Conclusions

This review has shown the great potential that the combination of MIPs with QDs offers to the development of optical sensors with unique advances in sensitivity and selectivity. MIP-QDs composites are categorized according to their chemical composition and an overview of the different strategies of synthesis has been described. Silica-based MIP-QDs sensors have received great attention since silica preserves the optical properties of QDs and also provides imprinted polymers with high thermal and mechanical stability. Additionally, well known and simple methods such as sol-gel and reverse microemulsion methods are typically used for their synthesis. Moreover, hybrid inorganic-organic MIPs-QDs sensors maintain the optical benefits of the silica-coated QDs, and simultaneously silica allows the attachment of the MIP. However, in some cases the silica is used as support material permitting the localization of the QDs at the surface or in the proximity of the surface, and therefore enhancing the fluorescence intensity. In both, hybrid inorganic-organic MIP and organic MIP based sensors, the recognition of target molecules is performed by an organic imprinted polymer whose composition is much more versatile due to the different functional monomers and cross-linkers that can be used.

Most of the applications described belong to the category of solution-based probes which have shown an outstanding performance for the analysis of water, food, plasma and urine samples. However, the development of MIP-QDs sensor microdevices, such as test strips and microfluidic paper-based analytical devices, has efficiently opened the possibility of performing reliable point-of-care testing and in-field diagnosis. Finally, MIP-QDs may represent a possible alternative to conventional monoclonal antibodies. Thus, promising developments have been done in targeting and bioimaging of tumor cells using MIP-QDs composites which are able to distinguish between healthy and cancer cells. Thanks to their excellent targeting ability, cancer treatments can be guided with cell imaging tools avoiding common side effects of traditional cancer therapy and a great activity in this field is expected in the coming years.
